# Chia, a large annotated corpus of clinical trial eligibility criteria

**DOI:** 10.1038/s41597-020-00620-0

**Published:** 2020-08-27

**Authors:** Fabrício Kury, Alex Butler, Chi Yuan, Li-heng Fu, Yingcheng Sun, Hao Liu, Ida Sim, Simona Carini, Chunhua Weng

**Affiliations:** 1 Columbia University in the City of New York, New York, NY United States; 2 New Jersey Institute of Technology, Newark, NJ United States; 3 University of California, San Francisco, San Francisco, CA United States

**Keywords:** Clinical trials

## Abstract

We present Chia, a novel, large annotated corpus of patient eligibility criteria extracted from 1,000 interventional, Phase IV clinical trials registered in ClinicalTrials.gov. This dataset includes 12,409 annotated eligibility criteria, represented by 41,487 distinctive entities of 15 entity types and 25,017 relationships of 12 relationship types. Each criterion is represented as a directed acyclic graph, which can be easily transformed into Boolean logic to form a database query. Chia can serve as a shared benchmark to develop and test future machine learning, rule-based, or hybrid methods for information extraction from free-text clinical trial eligibility criteria.

## Abstract (summary)

**Table:** 

Measurement(s)	Clinical Trial Eligibility Criteria • Analytical Procedure Accuracy
Technology Type(s)	digital curation • computational modeling technique
Sample Characteristic - Organism	Homo sapiens

Machine-accessible metadata file describing the reported data: 10.6084/m9.figshare.12765602

## Background & Summary

Clinical trial eligibility criteria specify rules for screening clinical trial participants and play a central role in clinical research in that they are interpreted, implemented, and adapted by multiple stakeholders at various phases in the clinical research life cycle^1^. After being defined by investigators, eligibility criteria are used and interpreted by clinical research coordinators for screening and recruitment. Then, they are used by query analysts and research volunteers for patient screening. Later, they are summarized in meta-analyses for developing clinical practice guidelines and, eventually, interpreted by physicians to screen patients for evidence-based care. Hence, eligibility criteria affect recruitment, results dissemination, and evidence synthesis.

Despite their importance, recent studies highlight the often negative impact these criteria have on the generalizability of a given trial’s findings in the real world^2,3^. When eligibility criteria lack population representativeness, the enrolled participants cannot unbiasedly represent those who will be treated according to the results from that study^4^. Given that eligibility criteria are written in free text, it is laborious to answer this representativeness question at scale^5^. A related challenge is to assess the comparability of trial populations, especially for multi-site studies: e.g.,, given two clinical trials investigating the same scientific question, can we tell if they are studying comparable cohorts? The manual labor required from domain experts for such appraisal is prohibitive. Another challenge is patient recruitment, or finding eligible patients for a clinical trial, which remains the leading cause of early trial termination^6,7^. Unsuccessful recruitment wastes financial investment and research opportunities, on top of missed opportunities, inconvenience, or frustration of patients when the clinical trial is terminated early or cancelled.

Computable representations of eligibility criteria promise to overcome the above challenges and to improve study feasibility and recruitment success^8^. The Biomedical Informatics research community has produced various knowledge representations for clinical trial eligibility criteria^9^, though nearly all of them predate the current state-of-the-art in machine learning, and some even predate contemporary electronic health records^9^. Early efforts to create annotated datasets in eligibility criteria have used a variety of methods including *ad-hoc* annotation^10^, manual annotation of standardized biomedical concepts^11^, as well as leveraging biomedical knowledge resources such as UMLS for automatic semantic pattern extraction^12^. The annotations in these datasets do not capture sufficient information to form the logical statements of a database query, and few annotated datasets are publicly available. Ross *et al*. published a dataset with 1,000 eligibility criteria and analyzed their semantic complexity, but the data were not amenable for machine learning^13^. 79 eligibility criteria were annotated by Weng *et al*. with semantic tags and relations, but these are too few to serve as a sufficiently large training resource^12^. The most robustly annotated and the only publicly available corpus to date was produced by Kang *et al*.^14^, who annotated eligibility criteria from 230 clinical trials, though all on Alzheimer’s Disease. Hence the corpus lacks generalizability to other diseases. These and other works have focused on bridging the gap between eligibility criteria and logical queries (Table 1), but the percentage of criteria that could be fully represented using these annotation models and used in database queries (here referred to as *criteria coverage*) is variable, ranging from 18% to 87%^5,14–18^.

**Table 1:** Annotated eligibility criteria with citations, methods of annotation, coverage, availability and size.

Citation	Annotation Method	Coverage	Availability	Criteria Count
Chondrogiannis *et al*., 2017^5^	Manual	87%	Online View Only	2,000
Tu *et al*., 2011 (ERGO)^15^	Manual	62%	Methods Only	1,000
Zhang *et al*., 2018^16^	Manual	85%	None	1,043
Milian *et al*., 2015^17^	Automated	18%	Methods Only	1,773
Lonsdale *et al*., 2006^18^	Automated	34%	Methods Only	1,545
Kang *et al*., 2017 (EliIE)^14^	Automated	71%	Available Upon Request	3,619
***Chia Annotation Model***	***Manual***	***86.8%***	***Publicly available***	***12,409***

A shared, sufficiently large dataset is much needed to boost machine learning natural language processing of eligibility criteria text. In this study we present *Chia*, a large annotated corpus of clinical research eligibility criteria from 1,000 diverse clinical studies. The annotations specify (a) the boundaries and semantic categories of named entities and (b) the Boolean operators needed to form the database query logic. As the first public large annotated corpus for clinical trial eligibility criteria, Chia can serve as a shared benchmark to develop and test future machine learning, rule-based, or hybrid methods for information extraction from free-text clinical trial eligibility criteria.

## Methods

### Chia’s Annotation Model (CAM)

The annotation model was developed by two annotators (FK and LHF), both with medical training, and one machine learning researcher (CY), following an iterative process. The entity categories are aligned with the domain names defined by the Observational Health Data Sciences and Informatics (OHDSI) OMOP Common Data Model (CDM), which is widely used in the medical research community for health data standardization^19^. Our annotation model is described in full in the Appendix. A brief description is provided below with a focus on its three main components: Entities, Relationships, and the resulting Annotation Graph.

#### Entities

Entities are concepts (e.g., *hypertension*, *platelet count*) and fall into one of the three types: *Domain, Field*, and *Construct. Domain* entities are the essential components of eligibility criteria, while *Field* entities and *Construct* entities are optional, depending on the semantic categories of *Domain* entities. Domain entities represent eight domains, i.e., observation, condition, person, device, drug, visit, procedure, and measurement. A *Field* entity represents a property of an applicable domain entity. For example, rule “Hemoglobin <8 g/dL” includes a *Domain* entity “Hemoglobin” and a *Field* entity “ <8 g/dL.” A *Construct* entity defines modifiers such as negation and repetition. For example, the *Negation* entity inverts the Boolean logic of the *Domain* entity being modified: *no history of heart disease*.

#### Relationships

Relationships express general Boolean algebra operators (AND or OR) between pairs of entities, as well as type-specific relationships (e.g.,, *has_value*, *has_temporal* etc.). This dataset represents 12 relationships: AND, OR, SUBSUMES, HAS_NEGATION (target argument is negation), HAS_MULTIPLIER (target argument is multiplier), HAS_QUALIFIER (target argument is qualifier), HAS_VALUE (target argument is value), HAS_TEMPORAL (target argument is temporal), HAS_INDEX (target argument is reference_point), HAS_MOOD (target argument is mood), HAS_CONTEXT (target argument is observation and not included in above relationships), and HAS_SCOPE (target argument is scope). The Boolean operator NOT, as mentioned in the previous section, is an entity instead of a relationship. When considering general Boolean relationships, both entities are independent, and the truth value of each term (i.e., is an entity “true”?) are resolved before applying Boolean logic to the relationship. For example, in “*Patients with diabetes or hypertension*,” “*diabetes*” and “*hypertension*” are independent *Domain* entities linked by an OR relationship between them.

#### Annotation graph

**Fig. 1:**
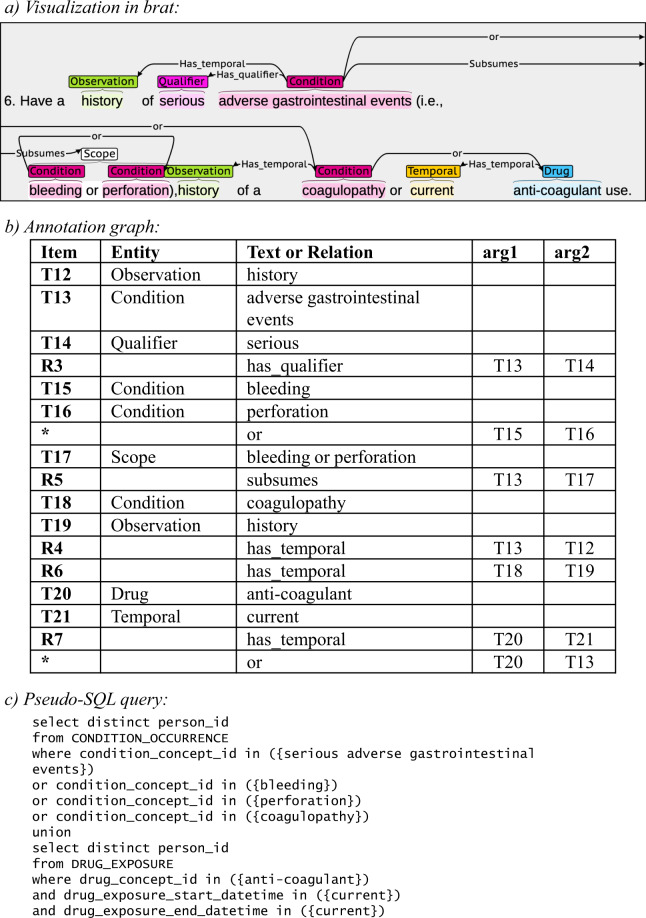
Sample eligibility criterion with associated visual annotation (**a**), annotation graph (**b**), and pseudo-SQL query for relational patient database (**c**).

The entities and relationships in each trial’s eligibility criteria can be represented as two *Annotation Graphs (AGs)* (one for inclusion criteria and one for exclusion criteria) to computationally represent the query logic, that is, how all entities and relationships should be used to construct an executable query. The entities form the nodes and the relationships form the edges in the graph, and each entity evaluates to true if matching data can be found to satisfy it. Parsing of a graph runs from root to leaf nodes, forming a single Boolean expression. If that expression evaluates to true when fed the data from one patient, it means such patient is eligible (if inclusion criteria) or is ineligible (if exclusion criteria). An example annotation graph can be seen in Fig. 1.

#### Scope objects

As eligibility criteria are used to identify patients eligible for a given trial, complex logic is often employed to ensure clinical judgments can be made with a high degree of accuracy. In some criteria, this complex logic can be considered ‘nested’ as a single entity is explained in greater detail and is provided with additional parameters whereby the entity could evaluate to true. An example is “*patient has hypertension (systolic blood pressure >135* *mmHg or diastolic blood pressure* >*85* *mmHg)*.” Here, the central entity is *hypertension*, which can be evaluated using the specified systolic and diastolic pressure ranges. We can treat both blood pressure measurements connected by an OR relationship as a single logical statement by labeling it a Scope object. Thus, this criterion can be rewritten to “*patient has hypertension, or [systolic blood pressure* > *135* *mmHg or diastolic blood pressure* > *85* *mmHg]*.” The *hypertension* entity is thus linked to this Scope object whereby satisfying either item results in the statement resolving to a single value of True.

### The annotation processes

#### Sampling of trials and loading into the annotation tool

We searched ClinicalTrials.gov on August 2, 2018 for actively recruiting, interventional (clinical trial), phase 4 studies, and obtained 2,913 trials, from which a random sample of 1,000 clinical trials was drawn. We focused on current studies as opposed to historic ones, assuming reporting quality is generally better in more current trials^20^, and prioritized phase 4 since they are more likely to be replicated via pure observational data analyses^21^. From each trial, a script downloaded and extracted every eligibility criterion (roughly defined as one line of free text), exported plain text files, and loaded them into the brat annotation tool (http://brat.nlplab.org/). That script is in R language and is available at https://github.com/WengLab-InformaticsResearch/CHIA.

#### Annotation of the eligibility criteria from the selected trials

The creation of Chia was performed by medical professionals (FK and LHF). Each annotator received a separate set of criteria loaded in brat^22^ and hand-created the entities and relationships as expressed above. In case of doubt for some concept, the annotator searched terminology at http://athena.ohdsi.org, which provides searching of concepts in the OMOP CDM. For the first 200 trials, both annotators regularly discussed adaptations to the annotation model based on their experience and re-annotated criteria as needed according to the changes being made. Once a satisfactory model was attained based on the consensus of both annotators, modifications were suspended and the task of annotation proceeded until the completion of the 1,000 trials. The final Chia dataset contains the summed and collectively revised work of the two annotators.

#### Post-processing the annotations

Minor post-production was performed to transform the ANN files produced by brat into a single long table in CSV format containing the entire dataset. That table also contains a number of variables that can be programmatically inferred from the annotations, e.g.,, which entities are roots in their annotation graphs. Additional post-processing was performed to generate two distinct datasets: one titled *With Scopes* and the other *Without Scopes* differing only in their utilization of Scope entity within the annotation model. Greater discussion of the reasoning behind the two distinct datasets is included in the Appendix, and all code used to generate these two models is available at https://github.com/WengLab-InformaticsResearch/CHIA.

To identify the target diseases of the 1,000 annotated trials, additional dataset enrichment was accomplished by leveraging the Aggregate Analysis of ClinicaTrials.gov (AACT) database^23^. This publicly available relational database contains all information about every study registered in ClinicalTrials.gov and is provided by the Clinical Trials Transformation Initiative (CTTI). The list of 1,000 unique NCT IDs included in our dataset was extracted and matched with their corresponding target conditions using the *conditions* table in the AACT database.

#### Parsing the annotation graph

A distinguishing feature of our dataset is its capacity to support the parsing of the entities and their relationships into a Boolean expression containing the logic of the database query that replicates the eligibility criteria of each clinical trial. A sample annotation alongside its associated annotation graph and pseudo-query is provided in Fig. 1.

## Data Records

The free-text of selected eligibility criteria, brat configuration files, and the annotated data files are all available on figshare at 10.6084/m9.figshare.11855817^24^. There are two folders of annotation files titled *With Scope* and *Without Scope*, describing the inclusion or exclusion of Scope entities (additional information in Appendix).

### Free-text (.txt) Files

Extracted free-text eligibility criteria from the 1,000 selected trials. Each text file adheres to the following naming format: *[NCT Number][Inclusion/Exclusion Status].txt*. Each row contains a single eligibility criterion.

### Annotation (.ann) Files

Non-post-processed annotations in brat’s native ANN output format. Each annotation file adheres to the following naming format: *[NCT Number]_ [Inclusion/ExclusionStatus].ann*. As per the ANN format, each line corresponds to a single entity or relationship, except for OR relationships as explained below. For entities, the fields are as follows: item ID (e.g., T1), entity type (e.g., Condition), string start index (e.g., 28), string end index (e.g., 55), text (e.g., ‘metastatic carcinoid tumors’). For relationships, the fields are as follows: item ID (e.g., R1), relationship type (e.g., *has_value*), root argument (e.g., T3), target argument (e.g., T2). For OR relationships, the second field lists the relationship type and subsequent columns list all items connected by this OR relationship. In order to visualize the annotations, one needs brat. Simply open, in brat, Chia’s entire unzipped folder. Instructions for downloading and installing brat are available at http://brat.nlplab.org/.

### Configuration (.conf) Files

These are the brat configuration files used to produce annotations following the definitions of CAM. They are used to load the.ann files into brat for viewing or editing them.

## Technical Validation

### Inter-annotator agreement

To evaluate the inter-annotator agreement of CHIA, we randomly selected 50 trials out of the 1,000 trials, constituting 5% of the whole dataset with 604 inclusion criteria and 1,034 exclusion criteria. The same 50 trials were provided to the two annotators (FK and AB) to annotate independently using the Brat annotation tool. To facilitate our agreement evaluation, the obtained raw annotations were converted into two formats: the CONLL-2003 BIO format^25^ and the token-level format with annotated label on each token. For example, in the CONLL-2003 BIO format, *Diabetes mellitus* is annotated as “diabetes B-Condition mellitus I-Condition”. In the token-level format, *Diabetes mellitus* is annotated as “diabetes Condition mellitus Condition”. An agreement was reached if both annotators annotated the whole span of *Diabetes mellitus* as Condition. These two formats enable us to evaluate both phrase-level agreement and token-level agreement rates. We calculated the Cohen’s kappa scores and Precision, Recall, and F1 as the measure of inter-annotator agreement. At the phrase-level, the Cohen’s kappa score is 0.8043 with Precision 81.39%, Recall 80.30% and F1 80.84. At the token-level, the Cohen’s kappa score is 0.8489 with Precision 81.67%, Recall 86.68% and F1 84.10.

### Dataset exploration and validation

Descriptive statistics were generated to report counts of entities, relationships, and combinations of both. A *coverage statistic* was used to compare this dataset with previous efforts. In an effort to assess the accuracy and immediate utility of individual annotations, the raw unprocessed annotated entities were mapped to standardized medical concepts. To do this we utilized an open-source concept mapping tool called Usagi (https://www.ohdsi.org/web/wiki/doku.php?id=documentation:software:usagi)^26^ to map free-text strings to medical concepts in the OMOP CDM and to provide a ‘mapping accuracy score’ based on string similarity that is automatically generated by the Usagi tool. All *Domain* entities were converted to lowercase and then input into Usagi v1.2.7 with the following restrictions: **(**1) ‘Filter standard concepts’ was unselected to allow for mapping to Standard and Classification Concepts, (2) ‘Filter by Domain’ was selected and the selected options included only the labeled Domain (e.g., *Condition*).

### Descriptive statistics

Chia contains 65,886 annotations for 12,409 inclusion and exclusion eligibility criteria from 1,000 trials targeting 1,130 unique diseases, with the most common one being Coronary Artery Disease (24 trials). 196 of the trials included healthy volunteers. 1,606 of the annotated eligibility criteria were determined as not being useful for patient-focused database queries for reasons highlighted in Table 2. Of those, 462 contained multiple entity annotations (e.g.,, “1. Signed informed consent” was annotated with *parsing_error, non-query-able*, and *informed_consent)* so the sum of annotations in Table 2 is greater than 1,606. 10,768 eligibility criteria had evaluable annotations, accounting for 86.8% of all the eligibility criteria (Table 1). When comparing it to the dataset prepared by Kang *et al*. in 2017^14^ (called EliIE), Chia is larger in size in terms of number of annotations, number of entity and relationship types, and criteria coverage. A top-level comparison of these datasets is shown in Table 3.

**Table 2:** Total count and percentage of unevaluable criteria using unevaluable entity annotations.

Entity Type	Count (%; n = 1,606)	Example
Non-query-able	557 (34.7%)	*In clinical judgement of study doctor, participant should not participate*
Post-eligibility	425 (26.5%)	*Unable to adhere to follow up schedule and treatment*
Informed_consent	223 (13.8%)	*Inability to give informed consent*
Pregnancy_considerations	172 (10.7%)	*Are not willing to use a reliable method of barrier contraception during the study*
Parsing_Error	135 (8.4%)	*3. Medications:*
Non-representable	120 (7.4%)	*Subjects who are incarcerated or wards of the state*
Competing_trial	86 (5.4%)	*Participation in other drug clinical trial within the last 4 weeks*
Context_Error	61 (3.8%)	*Hematologic laboratory values as outlined in the protocol*
Subjective_judgement	43 (2.7%)	*Viable tumor resection confirmed by two highly qualified surgical doctors*
Not_a_criteria	33 (2.1%)	*Screening tool: physical assessment (EKG), medical history*
Undefined_semantics	21 (1.3%)	*Presence of clinical contraindications for treatment with MTX*
Intoxication_considerations	5 (0.3%)	*Active alcohol or drug use or dependence which may interfere with adherence to study requirements*

**Table 3:** Comparison of EliIE and Chia Annotated Datasets.

Statistic	EliIE	Chia
Disease Domain	Alzheimer’s	Representative of all diseases
No. of Trials	230	1,000
No. of Criteria	3,619	12,409
No. of Annotations	15,596	65,886
No. of Entity Types	8	15
No. of Relationship Types	3	12
Criteria Coverage	71%	85.9%

Of the 41,699 entity annotations, 63.5% fall into the Domain category, 18.4% in the Field category, 17.5% in the Construct category and 0.4% were concepts unable to be annotated or deemed ‘unqueryable’ – additional information on these categories is included in the Appendix. *Condition* is the most common entity and *OR* the most common relationship. The most common relationship triplet is *Measurement-has_value-Value*; the second most common is *Condition-has_qualifier-Qualifier*. The most common relationship types are listed in Table 4 and the most common relationship triplets are listed in Table 5. OR relationships were not included in calculating the most common triplets as they do not always follow the *root-relationship-target* structure. 29.9% of OR relationships linked more than 2 entities and maximally linked 25 entities. All type-specific relationships contained the respective target entity type (e.g., *has_value* to Value entity) except for *has_temporal* (86.7% Temporal, 13.3% Observation) and *has_mood* (97.7% Mood, 2.3% Observation), though these different target types reflect the flexibility of the Observation Domain. For example, “history of” is considered an Observation despite its role in the *has_temporal* relationship. Concept mappings to the OMOP CDM with a score greater than or equal to 0.7 were considered strong matches. In total, 65.9% of raw annotated entities within the 8 Domain entity categories were considered strong mappings to the OMOP CDM. The percentage of strong mappings in each Domain category is shown in Table 6. Finally, the 15 most frequent tokens within the most common entity types are listed in Table 7 (fuzzy string-matching was used to generate token-level information).

**Table 4:** Most common relationship entities including overall count and percentage of all relationships.

Relationship	Count	Percent (n = 25,017)
OR	4,939	19.8%
has_value	3,806	15.2%
AND	3,679	14.7%
has_qualifier	3,535	14.1%
has_temporal	3,336	13.3%

**Table 5:** Most common relationship triplets (excluding OR relationships) including overall count and percentage of all relationship triplets.

Root Type	Relationship	Target Type	Count	Percent (n = 20,078)
Measurement	Has_value	Value	2799	13.94%
Condition	Has_qualifier	Qualifier	2445	12.18%
Condition	Has_temporal	Temporal	1323	6.59%
Temporal	Has_index	Reference_point	889	4.43%
Procedure	Has_temporal	Temporal	857	4.27%
Person	Has_value	Value	752	3.75%
Condition	AND	Drug	645	3.21%
Condition	Subsumes	Condition	624	3.11%
Drug	Has_temporal	Temporal	532	2.65%
Condition	AND	Procedure	514	2.56%
Procedure	Has_qualifier	Qualifier	465	2.32%
Condition	AND	Condition	459	2.29%
Condition	AND	Measurement	408	2.03%
Condition	Has_negation	Negation	380	1.89%
Procedure	AND	Condition	315	1.57%

**Table 6:** Mapping accuracy to OMOP CDM via Usagi per Entity Category.

Entity Category	Percent of Entities with Confidence Score ≥ 0.70
Condition	74.9%
Procedure	66.5%
Drug	64.8%
Device	62.1%
Person	61.8%
Measurement	55.2%
Observation	39.8%
Visit	31.3%

**Table 7:** Most common annotated entities by Domain.

Condition		Qualifier		Drug		Procedure	
Concept	Count	Concept	Count	Concept	Count	Concept	Count
pregnancy	442	severe	326	systemic corticosteroids	81	treatment	174
allergy	269	significant	117	medication	72	surgery	99
contraindications	197	active	114	anticoagulants	55	chemotherapy	81
infection	129	other	112	prednisone	49	radiation therapy	62
malignancy	104	uncontrolled	106	antibiotics	48	general anesthesia	58
hypertension	92	clinically significant	83	study medications	45	physical examination	42
lactation	90	chronic	57	antidepressants	40	cardiac surgery	41
heart failure	89	serious	55	aspirin	39	contraception	39
stroke	88	symptomatic	54	opioids	39	intubation	38
diabetes	82	moderate	47	vaccine	36	transplant	36
lactating	82	acute	43	statin	32	implantation	35
myocardial infarction	81	elective	40	warfarin	27	liver transplant	35
cardiovascular disease	64	untreated	39	insulin	27	dialysis	34
liver disease	63	stable	38	rifampin	27	hysterectomy	33
**Measurement**		**Person**		**Observation**		**Device**	
**Concept**	**Count**	**Concept**	**Count**	**Concept**	**Count**	**Concept**	**Count**
serum creatinine	77	age	577	breastfeeding	68	pacemakers	18
body mass index	65	female	355	life expectancy	64	intrauterine device	12
blood pressure	64	male	355	informed consent	29	prosthetic valve	12
weight	59	older	67	family history	18	prosthetic material	11
hemoglobin	57	adult	54	english speaking	16	prosthetic mesh	11
bilirubin	55	years	47	smoking	15	contraceptive implant	10
systolic blood pressure	52	children	32	childbearing potential	13	drug-eluting stent	9
diastolic blood pressure	52	patients	16	alcohol abuse	9	metal implants	9
pregnancy test	48	prisoners	13	evidence	8	device	8
platelet count	45	smokers	7	nursing	7	cochlear implants	8
creatinine clearance	44	infants	6	contraception	7	condom	7
ast [aspartate aminotransferase]	43	newborns	5	lactating	6	joint prosthesis	7
hba1c [hemoglobin a1c]	41	donor	5	last vaccination intervals	6	aneurysm clips	6
alt [alanine aminotransferase]	41	liver transplant recipients	5	suspected	6	metal in the body	6
asa [american society of anesthesiologists]	40	adolescents	5	sexually active	6	bare-metal stent	5

The Chia dataset contains a total of 4,161 annotated Scope objects with 1,009 having an incoming *subsumes* or *multi* relationship, serving a body of original and useful knowledge for electronic phenotyping (discussed further in Use Case 2 below). A few examples of Scope objects are presented in Table 8. The average number of entities contained within these Scope objects is 3.51 with a maximum of 82. Additionally, 2,318 *subsumes* or *multi* relationships are available throughout the dataset with that number increasing to 2,521 in the dataset without Scope objects (post-Scope decomposition).

**Table 8:** Examples of Scope objects in Chia (**contained on Scope object**).

Trial Number	Inc/Exc	Line	Sample Criterion
NCT02781610	Exclusion	5	…worsening lower respiratory symptoms (e.g.,, **pulmonary clean out, distal intestinal obstruction syndrome (DIOS), sinusitis**)
NCT02596555	Exclusion	13	…strong inhibitors of P-glycoprotein like **ketoconazole, cyclosporin, itraconazole or dronedarone**
NCT00650312	Inclusion	4	…judged normal and healthy during a pre-study medical evaluation (**physical examination, laboratory evaluation, 12-lead ECG, hepatitis B and hepatitis C tests, HIV test, and urine drug screen including amphetamine, barbiturates, benzodiazepine, cannabinoid, cocaine, opiates, phencyclidine, and methadone**)
NCT01373684	Exclusion	13	…immunodeficiency syndromes (e.g., **HIV positivity, auto-immune diseases, organ transplants other than cornea and hair transplant**)
NCT02531971	Inclusion	2	…including tobacco products (e.g., **cigarettes, cigars, chewing tobacco, gum, patch or electronic cigarettes**)

## Usage Notes

### Use case analyses

To demonstrate the utility of Chia, we propose two motivating use cases for this annotated corpus that can be explored in future research efforts.

#### Use Case 1: Machine learning model training for information extraction from eligibility criteria

Machine learning technologies can assist in parsing eligibility criteria. In previous research, Conditional Random Fields (CRFs)^27^, Convolutional Neural Networks (CNNs)^28^, Support Vector Machines (SVMs)^14^, hierarchical clustering^29^, distant supervision^30^, and other machine learning approaches have been used to extract entities and relationships from the free-text eligibility criteria to obtain structured representations. These extraction methods typically result in satisfactory accuracy but relatively low recall, such as the 94% accuracy and 18% recall described by Milian *et al*. in 2015^17^. There are a few possible explanations for this low recall. The first is that the training corpus lacks diversity in the types of eligibility criteria, as described in more detail in the Background section of this article. The second explanation is that most of the criteria (~85%) are very complex with various semantic patterns, including 35% of criteria containing more than one type of semantic pattern^13,31^. Finally, incomplete sentence structure and word ambiguity can also lead to extraction failures as described by Yuan *et al*. in 2019^32^.

**Fig. 2:**
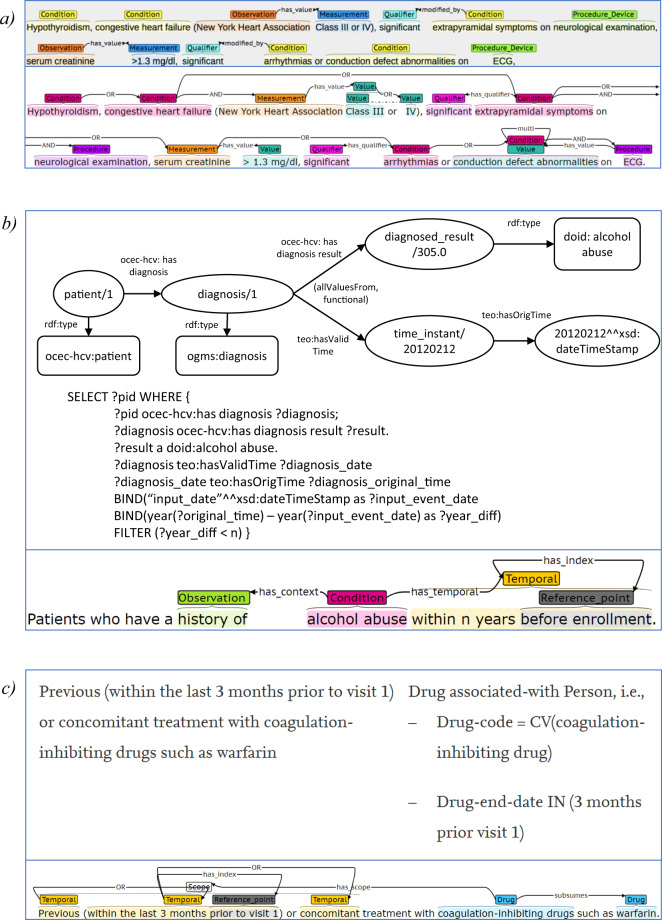
Comparisons of Chia annotation model to previous annotation efforts using identical sample eligibility criteria text. (**a**) EliIE annotation model proposed by Kang *et al*., (**b**) hepatitis C trials outlined by Zhang *et al*., (**c**) ERGO annotation model proposed by Tu *et al*.

A larger dataset of eligibility criteria with samples from a broad range of clinical trials is necessary to train a more accurate and robust extraction model. With 41,699 entities and 25,017 relationships annotated, Chia provides ample training data for machine learning research for identifying not only the boundaries and classes of named entities within medical text, but also the dependencies between these concepts. Further, when comparing to previous annotation efforts outlined in the Background section, the richness of the Chia model becomes clear as highlighted in Fig. 2. This direct criterion-to-criterion comparison allows for better appreciation of the increased level of connectivity between concepts (section A), simplicity in structure and format (section B), and improved granularity (section C) provided by Chia.

Chia’s non-flat annotation scheme is also noteworthy. Most corpuses for Named Entity Recognition (NER) training have adopted the flat annotation, disallowing discontinuous, nested, or overlapping entities^33^, whereas Chia represents them and supports the use case *Electronic Phenotyping* described below. Discontinuous and overlapping entities are required to capture coordination ellipsis, such as “*presence of pulmonary, hepatic or hematological cancer*.”, which is one type of ellipsis used in coordinating structure to achieve textual concision by omitting repeated words^34^. Coordination ellipses are more frequently used in medical language than in the general domain. More granular results are required for downstream tasks such as free-text based phenotyping. Existing annotated corpus containing overlapping entities are derived from biomedical literature, including GENIA and NCBI Disease corpora^35^. GENIA corpus is focused primarily on biological entities such as DNA, RNA, and protein^36^, and NCBI Disease is focused on disease mentions. To the best of our knowledge, Chia is the first clinical corpus of considerable size that is annotated in a non-flat fashion and supports annotation nesting and joining.

#### Use Case 2: Electronic phenotyping

Electronic phenotyping plays an essential role in disease knowledge discovery, application, and clinical research^2,37^. Electronic phenotyping supports cross-sectional and epidemiological studies, association studies, clinical risk factor analyses, and cohort identification^2^. In some cases, the phenotype definition is fairly simple (e.g., diagnosis of rheumatoid arthritis), but it can become more nuanced and complex (e.g., moderate or severe COPD exacerbation [requiring corticosteroids or increased dosage of corticosteroids and/or antibiotics or hospitalization]). Manual knowledge engineering to establish the linkage between the concepts in a phenotype is neither scalable nor efficient. Hierarchical relationships between annotated entities are explicitly defined in Chia via Scope objects, *subsumes* relationships and *multi* relationships, which provide reusable phenotyping knowledge. These annotations often indicate one concept (or group of concepts) that can be substituted for another because their meanings are inherently interconnected – oftentimes because the latter are clarifications or specifications of the former. For example, *Systolic Blood Pressure* > 130 and *Diastolic Blood Pressure* >85 can be used to define the condition *Hypertension*. There are 2,197 cases of subsumes in Chia, and Table 9 gives a few examples. Further, as eligibility criteria themselves serve to define a patient cohort, they can be considered to be small electronic phenotypes.

**Table 9:** Examples of subsumes relationships in Chia (**parent entity** and *subsumed entity*).

Trial Number	Inc/Exc	Line	Sample Criterion
NCT00050349	Inclusion	2	**…no major impairment of renal or hepatic function**, as defined by the following laboratory parameters: *total bilirubin < 1.5 X ULN; AST, ALT < 2.5X ULN ( < 5 X ULN if liver metastases are present)*
NCT00094861	Exclusion	7	**Presence or history of dysphagia or conditions predisposing to dysphagia** (e.g.,, *uncontrolled gastroesophageal reflux disease [GERD], dyspepsia, etc*.)
NCT00182520	Inclusion	2	…open label trial of one the following SRI’s…and demonstrating a **non or partial responses to SRI treatment** (*CGI-I of 3 or 4, Y-BOCS reduction of < 35%*)
NCT00343668	Exclusion	10	…significant **neurologic or psychiatric disorders** including *dementia or seizures*

## Supplementary information

Appendix

## Data Availability

All code used to generate and process the datasets described above is freely available at https://github.com/WengLab-InformaticsResearch/CHIA in the file titled *chia.R*. It was written in R version 3.3.3.
