# Supplementary material for: Chia, a large annotated corpus of clinical trial eligibility criteria
Source: Sci Data. 2020 Aug 27;7:281. doi: 10.1038/s41597-020-00620-0 (PMC7452886; doi:10.1038/s41597-020-00620-0)
Supplement: Supplementary file 1 — Appendix [file 41597_2020_620_MOESM1_ESM.docx]

Chia, A Dataset of Annotated Eligibility Criteria – Appendix: Chia’s Annotation Model

# Introduction

Chia's Annotation Model (CAM) is the annotation model designed and used to build the Chia dataset. Its objective is to model eligibility criteria into a computable database query, while simultaneously being economical in order to maximize the efficiency of the annotation task itself. CAM provides a conceptually-defined and unambiguous manner of assembling a database query from its annotations. The motivation underlying CAM, and the Chia dataset, is to train a machine learning algorithm capable of reading eligibility criteria in free text, as available from ClinicalTrials.gov, and generate their corresponding database queries.

A few notes on terminology

In order to allow for fluent discourse, the following terms are used extensively in this document and along project Chia. One annotation is either one entity or one relationship. In the case of a directed relationship, one side is the parent, the other the child, for example in A🡪B, A is the parent entity, B is the child entity, B is said to be downstream from A, A is upstream from B, and the relationship is said to be coming from A, or originating from A, and going to/towards B. Farther descendants and ascendants are also downstream and upstream, for example in A🡪B🡪C, C is also downstream from A. Each entity has a type (its label: Condition, Person, Measurement…) and a value (the free text annotated to the entity), while each relationship has a type (also its label: requires, subsumes, OR) and two arguments (the entities that are being connected). Annotation *types* are arranged into groups merely for discussion purposes.

# Problem definition and the workings of CAM

A patient is eligible for a particular study if they possess records evidencing that they satisfy all the specific eligibility criteria for that study at some specific window (range) of time. When this window of time occurs is irrelevant as long as all the criteria can be deemed to be simultaneously satisfied within it. For example, if a given eligibility criterion reads "Hemoglobin < 8 g/dL," an eligible patient must possess at least one record (one row in a SQL table) of a Hemoglobin measurement with a value below 8 g/dL at some point in their history. Conversely, an eligible patient must also *not* possess matching records satisfying any exclusion criteria within that same window; which is to say that the logics of the inclusion criteria are combined via Boolean AND logic, and the exclusion are combined via Boolean OR.

In order to construct the SQL query that tests for the presence of the needed records, one needs to know the tables, columns, values, and logical operators to be used. CAM specifies all those parameters by means of its entities and relationships, and an abstraction called the annotation graph (AG) which is the result of the interpretation of the former two. In CAM, each entity evaluates to *true* if the candidate patient possesses the record or records (table row[s]) satisfying that entity. However, for many entities, the truth value is contingent on another entity (or entities) due to the relationship between them, and together those entities evaluate to a single truth value. To reuse the example above, the criterion "Hemoglobin < 8 g/dL" is formed by the combination of one Measurement entity "Hemoglobin" linked to a Value entity "< 8 g/dL", which together evaluate to a single truth value *of true* (patient has a matching record in the MEASUREMENT table) or *false* (patient does not have a matching record).

From the truth values of the entities, the *annotation graph* then dictates how those entities should be combined into one Boolean expression representing all entities of a single eligibility criterion. Finally, as aforementioned, the inclusion criteria are linked via Boolean AND (∩), and the exclusion via Boolean OR (∪), which is then encapsulated by a Boolean NOT ($\boldsymbol{\neg}$), effectively producing a single Boolean expression for the entire body of eligibility criteria of a given study. See this example:

Inclusion:

- *Regular hemodialysis 3 sessions/week* [A]
  - $A_{1}$: hemodialysis
  - $A_{2}$: 3 sessions/week
  - $A = A_{1} A_{2}$
- *Recent angioplasty or coronary artery bypass grafting* [B]
  - $B_{1}$: Recent
  - $B_{2}$: angioplasty
  - $B_{3}$: coronary artery bypass grafting
  - $B = B_{1} {(B}_{2} B_{3})$
- *Men with age ≥ 18 years* [C]
  - $C_{1}$: Men
  - $C_{2}$: age ≥ 18 years
    - $C_{2.i}$: age
    - $C_{2.ii}$: ≥ 18 years
    - $C_{2}= C_{2.i} C_{2.ii}$
  - $C = C_{1} C_{2}=C_{1} (C_{2.i} C_{2.ii})$
- Boolean Inclusion expression (INC):

$$\boldsymbol{INC=A}\boldsymbol{B}\boldsymbol{C}\boldsymbol{=}\boldsymbol{A}_{\boldsymbol{1}} \boldsymbol{A}_{\boldsymbol{2}} \boldsymbol{B}_{\boldsymbol{1}} \boldsymbol{(}\boldsymbol{B}_{\boldsymbol{2}} \boldsymbol{B}_{\boldsymbol{3}}\boldsymbol{)} \boldsymbol{C}_{\boldsymbol{1}} \boldsymbol{(}\boldsymbol{C}_{\boldsymbol{2.i}} \boldsymbol{C}_{\boldsymbol{2.ii}}\boldsymbol{)}$$

Exclusion:

- *Intercurrent infections or surgery* [D]
  - $D_{1}$: Intercurrent
  - $D_{2}$: infections
  - $D_{3}$: surgery
  - $D = D_{1} (D_{2} D_{3})$
- *Sepsis* [E]
  - $\boldsymbol{E}_{\boldsymbol{1}}\boldsymbol{:}$ Sepsis
  - $\boldsymbol{E=E}_{\boldsymbol{1}}$
- *Receiving drugs affecting immune system* [F]
  - $F_{1}$: drugs affecting the immune system
  - $F = F_{1}$
- Boolean Exclusion expression (EXC):

$$\boldsymbol{EXC=D}\boldsymbol{E}\boldsymbol{F=(}\boldsymbol{D}_{\boldsymbol{1}} \boldsymbol{(}\boldsymbol{D}_{\boldsymbol{2}} \boldsymbol{D}_{\boldsymbol{3}}\boldsymbol{))} \boldsymbol{E}_{\boldsymbol{1}} \boldsymbol{F}_{\boldsymbol{1}}$$

Final Boolean expression (FBE):

$$\boldsymbol{FBE=INC} \boldsymbol{\neg EXC}$$

$$\boldsymbol{FBE= A}\boldsymbol{B}\boldsymbol{C}\boldsymbol{\neg}\left( \boldsymbol{D}\boldsymbol{E}\boldsymbol{F} \right)$$

$$\boldsymbol{FBE=}\boldsymbol{A}_{\boldsymbol{1}} \boldsymbol{A}_{\boldsymbol{2}} \boldsymbol{B}_{\boldsymbol{1}} \boldsymbol{(}\boldsymbol{B}_{\boldsymbol{2}} \boldsymbol{B}_{\boldsymbol{3}}\boldsymbol{)} \boldsymbol{C}_{\boldsymbol{1}} \boldsymbol{(}\boldsymbol{C}_{\boldsymbol{2.i}} \boldsymbol{C}_{\boldsymbol{2.ii}}\boldsymbol{)}\boldsymbol{\neg((}\boldsymbol{D}_{\boldsymbol{1}} \boldsymbol{(}\boldsymbol{D}_{\boldsymbol{2}} \boldsymbol{D}_{\boldsymbol{3}}\boldsymbol{))} \boldsymbol{E}_{\boldsymbol{1}} \boldsymbol{F}_{\boldsymbol{1}}\boldsymbol{)}$$

CAM has four groups of entities and two groups of relationships, as described below. As mentioned earlier, these groups are defined merely to facilitate discussion. The annotation graph is explained afterwards.

# Entity groups

## Domain

**Domain entities** represent semantic categories for a given concept. The types of entities included in this Domain group are outlined below with brief descriptions. Specific definitions for each of these domains are based on definitions provided by the Observational Health Data Sciences and Informatics (OHDSI) OMOP Common Data Model and more information can be found at <https://github.com/OHDSI/CommonDataModel/wiki>.

- Condition: the presence of a disease or medical condition stated as a diagnosis, a sign, or a symptom, which is either observed by a Provider or reported by the patient.
- Device: exposure to a foreign physical object or instrument which is used for diagnostic or therapeutic purposes through a mechanism beyond chemical action; includes implantable objects (e.g. pacemakers, stents, artificial joints), medical equipment and supplies (e.g. bandages, crutches, syringes), other instruments used in medical procedures (e.g. sutures, defibrillators) and material used in clinical care (e.g. adhesives, body material, dental material, surgical material).
- Drug: a biochemical substance formulated in such a way that when administered to a Person it will exert a certain physiological effect; includes prescription and over-the-counter medicines, vaccines, and large-molecule biologic therapies.
- Measurement: structured values (numerical or categorical) obtained through systematic and standardized examination or testing of a Person or Person's sample.
- Observation: clinical facts about a Person obtained in the context of examination, questioning or a procedure; includes any data that cannot be represented by any other domains, such as social and lifestyle facts, medical history, family history, etc.
- Person: demographic information used to describe a Person, including age, gender, race, ethnicity, etc.
- Procedure: activities or processes ordered by, or carried out by, a healthcare provider on the patient to have a diagnostic or therapeutic purpose.
- Visit: location or setting in which a Person is receiving medical services from one or more providers, including outpatient care, inpatient confinement, emergency room, and long-term care.

## Fields

**Field entities** represent properties of the Domain concepts. They may provide the value or range of values that must be present in a given lab test or timeframe for a previous diagnosis, and always appear downstream (even if indirectly) from at least one Domain entity.

- Temporal: represents a point in the line of time. Most often, a Temporal overlaps a *Reference_point* entity, and is linked to it via a *has_index*-type relationship (see definition of *Reference_point* below).
- Value: represents a structured value, either as a number (e.g. blood pressure *< 140/90 mmHg*) or as a concept (e.g. *elevated* serum creatinine). When specifying a number value, the only components accepted inside its free text are (extending the above example): logical operator (*<*), numeral (*140/90*), unit of measure (*mmHg*).

## Constructs

**Construct entities** serve syntactic purposes. Like Fields, they require a relationship to another entity to form any meaning. With the exception of Scope, all Construct entities are necessarily children of the entity whose meaning they complement or modify.

- Scope: distributes all relationships coming to it, and departing from it, to all roots of annotation graphs included in it. The critical purpose of the Scope is to allow the creation of layered logic (e.g., A and (B or C)), but it is also used as a shortcut to expedite the annotation task when multiple entities are connected in "combinatorial" fashion, such as multiple Qualifiers being all applied to multiple Conditions. To allow for multiple use cases of the Chia dataset, one model will include SCOPE objects and another will not. Further discussion of this is provided in the “Discussions about CAM” section.
- Negation: provokes a Boolean negation on its parent entity. If the truth value of the parent evaluates to *false*, it then becomes *true*, and vice-versa.
- Qualifier: subsets the meaning of its parent by imposing a further constraint. The value of a Qualifier oftentimes serves as a supplement to the value of its parent, that is, it may be the case that the free text contained by a Qualifier can be concatenated with the free text contained by its parent (e.g. a Condition) to form one string that can then be linked to a single code. For example, if the free text reads "familial diabetes insipidus", we might have one Condition "diabetes insipidus" linked to one Qualifier "familial." Another common case is for Qualifiers to express the anatomic location of a Condition (e.g. *facial* trauma) or the severity of a Condition (e.g. *severe* renal impairment).
- Multiplier: specifies either dosage of a Drug entity, or repetition type of entity (e.g. "at least two of...").
- Reference_point: Always comes downstream (usually directly) from a parent *Temporal*, and specifies a concept whose timestamp anchors that *Temporal*. For example, in "within two weeks of a blood transfusion" this entire text string is one *Temporal*, and it contains (overlaps) the *Reference_point* "blood transfusion."
- Mood: transforms the meaning of its parent into a different kind of statement that is not about the literal presence of the parent. For example, in "eligible for surgery" the Mood "eligible for" denotes that satisfying this criterion does not require the presence of records of the surgery, but rather the presence of concept(s) associated to that surgery – in this case, the patient's *eligibility* for it.

# Relationships

The relationship types of CAM are derived from Boolean algebra—AND, OR and NOT—to which we add the consideration of whether the meaning of the entities depend on each other or not. For example, in "Hemoglobin < 8 g/dL", the meaning of the two entities (Measurement "Hemoglobin" and Value "< 8 g/dL") depend on each other and need to be resolved in the context of each other; while in "Pregnant women with abdomen discomfort" the three entities (Condition "Pregnant", Person "woman" and Condition "abdomen discomfort") are independent and therefore have their own truth values (*true* or *false*).

Notice that this notion of dependence/independence is *not* based on medical or domain-specific knowledge (e.g., "the abdomen discomfort is being caused by the pregnancy, and pregnancy can only possibly happen among women"), but only on how the data elements need to be specified for the purpose of assembling a SQL query. In "Hemoglobin < 8 g/dL", the query seeks to locate a single row in the corresponding table containing simultaneously the Measurement "Hemoglobin" and a Value “smaller than 8 g/dL.” In "Pregnant women with abdomen discomfort", the query seeks three separate rows: one for a Condition of “Pregnant,” one for the Person of “woman,” and a third for the Condition of “abdomen discomfort.” In the context of the latter example, oftentimes the relatedness of these separate rows is established based on their timestamps; in other cases, a literal join (SQL join) is required. This matter is discussed later in the section *Time windows, row linkage and record relatedness.*

The relationships that establish semantic dependency are called conditional in CAM and are differentiated based on the type of entities they connect:

1. AND
2. OR
3. SUBSUMES
4. HAS_NEGATION (target argument is *negation*)
5. HAS_MULTIPLIER (target argument is *multiplier*)
6. HAS_QUALIFIER (target argument is *qualifier*)
7. HAS_VALUE (target argument is *value*)
8. HAS_TEMPORAL (target argument is *temporal*)
9. HAS_INDEX (target argument is *reference_point*)
10. HAS_MOOD (target argument is *mood*)
11. HAS_CONTEXT (target argument is *observation*)
12. HAS_SCOPE (target argument is *scope*)

Again, a conditional relationship signifies that the meanings of the entities depend on each other and resolve to a single truth value, while non-conditional relationships signify that the entities have their own decidable truth value that is ascertained before applying the Boolean logic of the relationship. For example, a Measurement entity is always linked via *has_value* to a Value entity, which means that Value is the result of the Measurement. Multiple Conditions are commonly linked via OR or AND (non-conditional), or can be linked via the conditional *subsumes* (e.g. “gastrointestinal events such as upper GI bleed or gastric ulcer perforation”).

The following adaptations were made to the resulting twelve relationship types for the purpose of expediting the annotation process:

1. In the absence of any other relationship between any two *annotation graph roots* (entities with no parents; explained later in this document), the AND is the default and always present. This effectively makes the AND unnecessary, so it never explicitly appears and is, in practice, absent from the annotations, even though its effect as the default relationship remains active.
2. In order to specify which words represent negation, the NOT relationships were replaced by the Negation entity, which exerts the effect of a Boolean negation on its parent entity which is linked via HAS_NEGATION. The value of the Negation entity is the piece of free text that produces the meaning of negation, i.e., it is the word or group of words that, if removed, would make the negation cease to exist.

Finally, a special type of relationship called multi was added to enable multi-labeling, that is, one piece of free text relating to more than one entity type. By definition, the multi relationship is strictly a *bonus* intended to provide additional information for the purpose of helping disambiguate the meaning of an entity (i.e., aid the task of Named Entity Linking). A multi relationship, as well as any entity or entities appearing downstream from it, can be ignored and still be left with a valid and complete annotation of the criterion in its full query logic.

The special optional attribute

Attributes are Boolean flags that an entity can have, and CAM contains only one, called optional, whose default value is *false*. If an entity has this attribute (i.e. its value is *true* for that entity), the entity does not need to be satisfied and everything coming downstream from it can also be ignored, i.e., can be removed from the final Boolean expression that represents the criterion. If, however, the entity flagged as optional is satisfied on its own (evaluates to *true*), then everything downstream from it must also be satisfied, otherwise the flagged entity will revert back to *false*. Notice that the optional, therefore, transforms the entity into a logical gate akin to an “if” statement in a programming language: “If” the entity is satisfied, then its descendants must be satisfied as well. This feature is mostly employed in a mechanism called Call-and-return, explained later in this document.

# Annotation Graph

The Annotation Graph (AG) represents the query logic, which is effectively one Boolean expression. All eligibility criteria from one trial, together, form one AG whose evaluation determines whether a given patient is eligible or not. Each Entity can evaluate to False or True according to the patient's data, and the edges of the AG specify how to combine those truth values into a final result of *true* (patient is eligible) or *false* (patient is not eligible).

The parsing (traversal, evaluation) of the AG is done by executing the logical operations specified by the Relationships using the truth values of the Entities. The AG is parsed from the roots (specified below) to the leaves. After processing the combined Boolean logic of the root and all its children, the processing follows to the Boolean logic of the non-conditional relationships between the roots. As aforementioned, in the absence of any other relationship, there is always an AND relationship linking all roots.

Roots in the annotation graphs

The roots are the entities not descendant of any other, nor horizontally linked to any entity that descends from another one. In other words, imagine that you start at “level” 1, and each conditional relationship increases (“goes down”) the level by 1 while the non-conditional relationships maintain the current level – the roots are the entities that are still at level 1 after all relationships have been considered.

Special constructs in the annotation graphs

In a few cases, specific entities exert predefined semantic effects on their parents. The most common case is the Negation entity, which exerts a simple Boolean negation on the final truth value of its parent entity (after all other processing of the parent has already been completed).

# Discussions about CAM

## The interplay of CAM and Named Entity Linking

CAM specifies the entities, the relationships, and how they can be combined into a query; but it declares out of its scope the task of linking the entities to their normalized versions, i.e. the codes they represent, the task of *Named Entity Linking*. This "gap" between *representation* and *implementation* has for long been known in the Informatics literature as the curly braces problem.

In the example "Hemoglobin < 8 g/dL," CAM specifies that "Hemoglobin" belongs to the Measurement domain, and that one needs to query the MEASUREMENT table for rows containing the *concept corresponding to "Hemoglobin,"* but CAM does not specify which exact concepts or strings should be used. Indeed, given solely the free text available from the protocol of a clinical trial, which in this example is nothing more than the word "Hemoglobin", the task of specifying what are its correct corresponding codes in a given data model is an open, unsolved challenge belonging to the research field of Named Entity Linking (NEL). Even in such a simple example as "Hemoglobin", a search in the OHDSI Common Data model for this term returns 862 codes in the Measurement domain alone, even after filtering for standard and valid concepts (<http://athena.ohdsi.org/search-terms/terms?domain=Measurement&page=1&pageSize=15&query=hemoglobin>). Given the paucity of detail provided by the free text, performing NEL to any practical, usable degree is only possible if resorting to auxiliary methods such as preferred codes, ranking eligible matches, code prevalence-based preferences, and others as explored in the NEL literature.

While performing NEL is beyond the scope of CAM, and an unsolved scientific challenge, we must still observe that, in devising an annotation model capable of translating free text into a database query, there exists an intrinsic interplay between the annotation model and the task of Named Entity Linking. Defining how to approach this interplay is required for defining the boundary between the annotation model and the task of NEL, that is, it is required for defining the annotation model. This is explained below.

Absent of a definition on how to approach, i.e. how to draw the conceptual line separating the annotation model from the task of NEL, the task of modeling a database query that represents a given eligibility criterion could be relegated to the task of Named Entity Linking (NEL). This is because one could create a single entity that wholly defines the criterion. Consider this example:

Patient is hemodynamically stable with hemoglobin >10 g/dL.

It is possible that there exists a single entity, which, if satisfied, means that the patient is eligible:

{Patient is hemodynamically stable with hemoglobin >10 g/dL.}: If this entity is satisfied, the patient is eligible.

Whoever (or whichever algorithm) is responsible for performing NEL would still be left with a difficult challenge. *Therefore, at its most fundamental aspect, the purpose of the annotation model must be to subtract complexity from the task of NEL.* See the example again, but annotated differently:

Patient is {hemodynamically stable} with {hemoglobin} {>10 g/dL}.: This facilitates NEL -- the entities are "*hemodynamically stable*", "*hemoglobin*" and *">10 g/dL*". If these three entities are satisfied, the patient is eligible.

While the example above probably seems trivial, the nature of medical knowledge easily blurs the lines between entity boundaries and domains, and often there exist multiple valid ways to represent the same piece of free text. See the examples below:

Patient is HIV-positive.

- Is "HIV-positive" the name of a Condition? Or is "HIV" the name of a test, and "positive" its result? Considering that multiple valid codes exist for all these alternatives, how should the SQL query be assembled?

Patient has untreated diabetes mellitus type 2.

- Is "*untreated diabetes mellitus type 2"* the name of a Condition? Or is "untreated" a negated version of "treated", that is, the absence of the Procedures and/or Drugs that are used for diabetes?

## To Scope or Not to Scope

Complex logic is often employed in eligibility criteria to ensure clinical judgments can be made with high accuracy and precision. In many criteria, this complex logic can also be considered ‘nested’ as a single entity may be conditionally linked to a separate logical statement. Consider the example below:

Patient has high blood pressure (systolic blood pressure >= 140mmHg or diastolic blood pressure >= 90mmHg).

In this example, the condition entity *high blood pressure* can be satisfied by a previous diagnosis of high blood pressure/hypertension or a recent blood pressure measurement of >= 140 systolic or >= 90 diastolic. In this setting, it is helpful to treat the entities and values inside the parentheses as a single logical statement which is accomplished by labeling it as a SCOPE object. Then, the *high blood pressure* entity is linked to this SCOPE via a *subsumes* relationship such that satisfying either item will result in the statement resolving to TRUE.

Though the SCOPE is a useful setting of eligibility criteria annotation, it also poses some unique challenges. Chief among them is the difficulty it introduces in training machine learning models to accurately parse real-world criteria. The concept of a SCOPE is highly subjective and often relates to the context of the entity and criteria around it. Given the human component in building these objects, it is likely to be very difficult for a computer-enabled system to accurately and consistently identify SCOPE objects within criteria and connect them to the correct entities. To avoid this dilemma, we employed a relatively simple means of ‘Scope Decomposition’ to remove SCOPE objects within our annotation model while maintaining the same logical information.

To perform this decomposition, the *root(s)* of each scope are selected as the representative(s) of the relationship(s) that such scope is involved with. If a scope object originates a relationship, then the root(s) of this scope will originate the same type of relationship after the scope decomposition. For the presence of multiple roots for this scope, each root will originate a relationship connecting to the same target respectively. Similarly, if a scope object is the target of a relationship, then the root(s) of this scope will become the target(s) that this relationship is connecting to. For the presence of multiple roots for this scope, a relationship originated from the same entity will connect to each root respectively. For instance, two relationships (“*high blood pressure”* SUBSUMES “*systolic blood pressure”*) and (“*high blood pressure”* SUBSUMES “*diastolic blood pressure”*) are obtained after decomposing the scope object in the above example.

To select a root of a scope object, we follow the same principle that is used to determine the root(s) of an annotation graph, i.e., a root should be an entity that is at top level after all relationships have been considered in this scope. Every entity/scope in a scope object will be scanned for the number of incoming and outgoing relationships it has. The entities/scopes with the least incoming relationship(s) will become root candidates. If an outgoing relationship is present for any root candidates, all candidates are roots. For instance, the scope object in the above example has two entities “*systolic blood pressure”* and “*diastolic blood pressure”* with no incoming relationship, but two outgoing relationships *has_value* to “*>= 140mmHg”* and “>= 90mmHg”, respectively. Thus, both entities “*systolic blood pressure”* and “*diastolic blood pressure”* are the roots of this scope object. In the case of no outgoing relationship existing for all root candidates, the first candidate appearing in the text will be used as a root, and all other candidates will be connected via AND relationships.

A scope object can have one or more roots. A root of a scope can only be an entity. Thus, if a nested scope is selected as a root candidate, the same rule is applied to decompose this scope until an entity root is found. For nested scope decomposition, the decomposition is processed from the outermost to the innermost scope.

The code used to perform this *SCOPE decomposition is* provided alongside all other code used in this analysis. Additionally, two separate annotated datasets are being released with this model: *With Scopes* and *Without Scopes*. Both contain the same logical information, but the *Without Scopes* dataset is likely to be more amenable to computer-enabled systems where the *With Scope* dataset will contain the original SCOPE object.
